# The Objection Answered

**Published:** 1910-06-04

**Authors:** 


### THE OBJECTION ANSWERED.

Sir,—I have examined once more the prices and qualities of dressings which were placed at my disposal for the purpose of the article on "Surgical Dressings" which appeared in your issue of May 7, to which the Liverpool Lint Co. takes exception, and I can confirm them as being strictly accurate, with the exception of one item, which, I regret to say, was incorrectly copied from the rough original, and the error remained undetected. Absorbent wool should have been quoted at 6½d. per lb. instead of 6d. I desire, therefore, to amend this quotation to the figure now given. The previous contract was 6d., but the present supply, the sample of which I have before me, is 6½d. per lb.

The prices were the result of careful selection from a number of firms of high repute, and dressings are being supplied in a very satisfactory manner at the prices quoted, and are giving satisfaction in use.

I notice that the Liverpool Lint Co. itself is included in the schedule I have before me as supplying some of the goods at the prices quoted, and one item at less than the price quoted, and I am assured by the purchaser that the qualities of these goods are excellent for the prices charged, and that experience has taught that it is more economical to use these dressings than more expensive qualities.

Yours faithfully,
The Writer of the Article.

